# Samarium Diiodide Acting on Acetone—Modeling Single Electron Transfer Energetics in Solution

**DOI:** 10.3390/molecules27248673

**Published:** 2022-12-08

**Authors:** Luca Steiner, Andreas J. Achazi, Bess Vlaisavljevich, Pere Miro, Beate Paulus, Anne-Marie Kelterer

**Affiliations:** 1 Institute of Physical und Theoretical Chemistry, NAWI Graz, Graz University of Technology, Stremayrgasse 9, 8010 Graz, Austria; 2 Institut für Chemie und Biochemie, Freie Universität Berlin, Arnimallee 22, 14195 Berlin, Germany; 3 Physikalisch-Chemisches Institut, Justus-Liebig-Universität Giessen, 35392 Giessen, Germany; 4 Department of Chemistry, University of South Dakota, 414 E. Clark St., Vermillion, SD 57069, USA

**Keywords:** computational chemistry, lanthanoide chemistry, density functional theory, ketyl radical, HMPA influence, reduction potential, effective core potential

## Abstract

Samarium diiodide is a versatile single electron transfer (SET) agent with various applications in organic chemistry. Lewis structures regularly insinuate the existence of a ketyl radical when samarium diiodide binds a carbonyl group. The study presented here investigates this electron transfer by the means of computational chemistry. All electron CASPT2 calculations with the inclusion of scalar relativistic effects predict an endotherm electron transfer from samarium diiodide to acetone. Energies calculated with the PBE0-D3(BJ) functional and a small core pseudopotential are in good agreement with CASPT2. The calculations confirm the experimentally measured increase of the samarium diiodide reduction potential through the addition of hexamethylphosphoramide also known as HMPA.

## 1. Introduction

Samarium diiodide is a versatile yet selective one-electron donating agent. It was introduced in 1977 by H. B. Kagan and is thus known as “Kagan’s reagent” [1,2]. Over the past 30 years, it has found widespread applications in organic synthesis [3,4,5,6,7,8,9,10,11,12,13,14].

Nevertheless, there is a lack of understanding regarding its reactivity and simultaneous selectivity at the atomic level [4,6,7,8,9,10,15]. Theoretical studies have tried to fill this gap over the last 15 years [16,17,18,19]. However, the high weight (relativistic effects) and open f shell character of the septet state of samarium make calculations challenging. The explicit treatment of the first solvation shell is important for the steric outcome of reactions [16]. The sevenfold coordinated solvent shell in tetrahydrofuran (THF, $SmI_{2}{\left(\mathrm{THF}\right)}_{5}$) leads to a large number of atoms involved in $SmI_{2}$-mediated reactions. In 2010, Maron and coworkers simulated reactions of $SmI_{2}$ with density functional theory (DFT) by replacing samarium with europium [20,21]. The widely used DFT software at the time could not easily handle even numbers of f electrons in the core. The europium atom was represented by a large-core effective core potential (ECP) that included all 4f electrons in the core. Since then, the field has switched to use actual samarium. It is mainly represented by the two large-core Stuttgart–Dresden ECPs [18,19]. One of them has all six 4f electrons in the core [22,23]. Hence, it can only represent Sm${}^{2+}$. The other ECP has five 4f electrons in the core [22,23]. The last 4f electron is explicitly included in the calculation, and the ECP is optimized to represent Sm${}^{3+}$. Even the most recent studies often employ these two large-core ECPs [19,24]. In the reduction step, samarium switches from Sm${}^{2+}$ to Sm${}^{3+}$ and donates one electron. The large-core ECPs cannot represent this key step of the $SmI_{2}$-mediated reactions. Some groups [16,17,25], including us, have switched to calculating all reaction steps with the small-core Stuttgart–Dresden ECP [26]. It has 28 electrons in the core, and all the 4f electrons are explicitly included in the calculation. However, these DFT calculations with the small-core ECP show that the reaction step from Sm${}^{2+}$ to Sm${}^{3+}$ is energetically unfavorable [16,25]. This is contrary to the experiments. $SmI_{2}$-mediated reactions are usually performed under mild conditions with high yields [10,27]. The divergence between experimental and theoretical results could be caused by the approximations made in common DFT. Maron and coworkers made a first step by investigating the single electron transfer (SET) by $SmI_{2}$ with the complete active space self-consistent field (CASSCF) method for benzophenone and related systems [17]. They concluded that the density functional method (B3PW91 in their case) gives a wrong description of the SET reaction of $SmI_{2}$, but the SOMO-LUMO gap approach can qualitatively describe the energetics.

The present study takes the validation of the correct theoretical method for $SmI_{2}$ reductions one step further. Acetone (ACE) with $SmI_{2}{\mathrm{THF}}_{4}$ represents the smallest model system for the important $SmI_{2}$-mediated ketyl coupling [27]. The ketyl radical is postulated to be in equilibrium with unreduced carbonyl groups [28]. The *acetone-ketyl equilibrium* is on the side of the acetone molecule. The presented study utilizes scalar relativistic all-electron CASPT2 to calculate the reduction of ACE by $SmI_{2}$ modelled in THF by explicit solvation. A DFT functional comparison shows that PBE0-D3(BJ) and the small-core Stuttgart–Dresden ECP accurately reproduce the CASPT2 energy.

Hexamethylphosphoramide (HMPA) is a common additive for $SmI_{2}$-mediated couplings [10,29,30]. HMPA as a cosolvent replaces THF in the first solvation shell of $SmI_{2}$ [31,32]. Furthermore, the SmI bond increases, and apparently the iodide molecule is replaced by THF or HMPA, resulting in solvent-separated ion pairs $\left[Sm{\left(\mathrm{HMPA}\right)}_{4}{\left(\mathrm{THF}\right)}_{2}\right]I_{2}$ at low HMPA concentrations or $\left[Sm{\left(\mathrm{HMPA}\right)}_{6}\right]I_{2}$ for HMPA concentrations higher than 10 equivalents, respectively [33]. In experimental studies, an increase in the reduction potential in dependence of the HMPA concentration was measured. Reported influence ranges from 36 kJ/mol (kinetic measurements) [28] up to 69.5 kJ/mol (linear sweep experiment), while the latter study investigates concentrations of up to 6 equivalents of HMPA [34]. We model the influence of the computational demanding HMPA explicitly in DFT calculations. The reproduction of the experimental trends of the influence in dependence of the HMPA concentration shall further validate the recently used methodology [16].

## 2. Results

### 2.1. Optimized Structures

**Figure 1:**
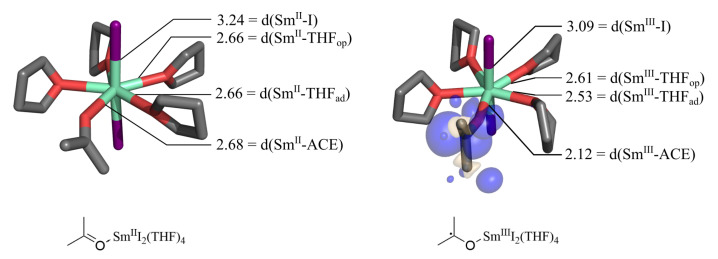
ACE-Sm^II^$I_{2}$${\left(\mathrm{THF}\right)}_{4}$ (**left**) is optimized with PBE0-D3/def2-TZVP (I, C, O, H) and ECP52MWB-II (Sm), while ECP51MWB-SV (Sm) converges to the ACE${}^{•}$-Sm^III^$I_{2}$${\left(\mathrm{THF}\right)}_{4}$ structure. Spin difference density of the doublet (**right**) is shown for a density value of the isosurface of 0.002 au. Bond distances (d) are printed in (Å). Important bond angles are ACE-Sm^II^$I_{2}$: ${∡}_{ISmI}=$176.6${}^{\circ }$, ${∡}_{SmOC}=$142.1${}^{\circ }$; ACE${}^{•}$-Sm^III^$I_{2}$: ${∡}_{ISmI}=$170.6${}^{\circ }$, ${∡}_{SmOC}=$164.2${}^{\circ }$.

The samarium diiodide-mediated SET was modelled in an explicit solution by the ACE-Sm^II^$I_{2}$${\left(\mathrm{THF}\right)}_{4}$ and ACE${}^{•}$-Sm^III^$I_{2}$${\left(\mathrm{THF}\right)}_{4}$ system shown in Figure 1 by the use of two different large-core pseudopotentials for Sm. Kefalidis et al. describe the strategy in great detail [17,35]. With the 4*f* electrons in the core, the ACE-Sm^II^$I_{2}$${\left(\mathrm{THF}\right)}_{4}$ system is a closed-shell system, and the ACE is bound in its electronic ground state configuration to Sm. The Sm-ACE distance is quite long, at 2.68 Å, which indicates a weak bond. This is different in the case of ACE${}^{•}$-Sm^III^$I_{2}$${\left(\mathrm{THF}\right)}_{4}$, where the ECP51MWB models Sm^III^. The one explicitly treated f-electron of the Sm is readily transferred to acetone, forming a ketyl radical. The Sm-ACE bond length significantly shrinks to 2.12 Å. The bond angle ${∡}_{SmOC}$ increases significantly by 22.1 ${}^{\circ }$, which can be related to both a cause and a result of the bond length shortening of Sm-ACE. This can possibly be traced to the change of the proportion between the covalent bonding and the electrostatic attraction. The antibonding $\pi^{*}$ orbital of ACE is filled with an electron in ACE${}^{•}$-Sm^III^$I_{2}$${\left(\mathrm{THF}\right)}_{4}$, which will be further discussed. Bond shortening upon electron transfer is expected due to the oxidation-induced contraction of the electron cloud around Sm^III^, which is further discussed in the literature [36]. The SET-induced Sm-ACE bond length shortening causes a polarizing effect, which leads to anisotropic bond length changes in the surrounding THF ligand. Changes in the bond lengths of Sm-I are greater than changes in THF because iodine is already charged and attractive forces rise from the SET. Hence, the SET leads to a tighter binding situation through increased electrostatic attraction and bond formation.

Note that the acetone substrate is not planar after SET but slightly transformed into a trigonal pyramidal conformation. This is reasonable since the occupied $\pi^{*}$ orbital enforces a partial $sp^{3}$ character. The density of the ketyl radical requires more volume due to the distortion from partial $sp^{3}$-hybridisation and the additional electron. A detailed comparison between this system and the unbound $\mathrm{ACE}+e^{-}\to$ACE${}^{•-}$ couple is found in the Supplementary Materials SI.1.

### 2.2. Electron Transfer Reaction Energy

Single-point energies for the PBE0-D3-optimized structures ACE-Sm^II^$I_{2}$${\left(\mathrm{THF}\right)}_{4}$ and ACE${}^{•}$-Sm^III^$I_{2}$${\left(\mathrm{THF}\right)}_{4}$ are calculated by CASPT2 and density functional theory in this section (cf. Table 1). The CAS result states that the equilibrium is on the side of acetone. The energy of the SET is 48.94 kJ/mol according to the CASPT2(6,13) septet calculation. The energy difference takes into account the first solvation shell (by THF), static and dynamic correlation and scalar relativistic effects. Thermal corrections, spin orbit coupling and the second solvation shell are not taken into account. Correction from perturbation theory is only 3.00 kJ/mol, which indicates that the CASSCF(6,13) accounts quite well for the dynamic correlation. An initial active space consisting of the seven 4f orbitals and the ligand orbital of interest was selected, along with the appropriate number of electrons resulting in a CAS(6,8). The smaller active space is further discussed in the Supplementary Materials SI.2. However, calculations with an additional five correlating orbitals were performed. If one thinks in terms of the Sm atomic orbitals, these five orbitals were expected to represent the 5*d* shell. During orbital optimization, higher angular momentum functions are mixed in the molecular orbitals, as is often the case. This larger (6,13) active space resulted in reasonably large changes in the triplet-quintet spin splitting energies compared to the smaller space, and for this reason, only the larger active space is reported.

The SET energy results for the DFT benchmark are shown in Table 1. The non-hybrid functionals PBE and TPSS predict lower energies than their hybrid analogues. The meta-GGA functionals TPSS and TPSSH tend to be too low in energy. The B3PW91 functional is used without dispersion in recent studies and delivers good energies only if dispersion correction is not applied. The functionals PBE0-D3 and B3LYP-D3 show similar ET-energies compared to CASPT2. Results for the already mentioned functionals are further discussed in the Supplementary Materials SI.3, together with occupation numbers and partial charges. However, PBE0-D3 is slightly closer in energy to the CASPT2 value and is further discussed in the upcoming section.

### 2.3. Comparison of Quintet and Septet Spin State

Not only can the high spin (septet) ground state be involved in the SET, but other spin states like the quintet may also contribute. Therefore, we investigate different spin states and their ability to transfer an electron for the given PBE0-D3 structures at both the CASPT2 and the PBE0-D3 level septet and quintet.

**Figure 2:**
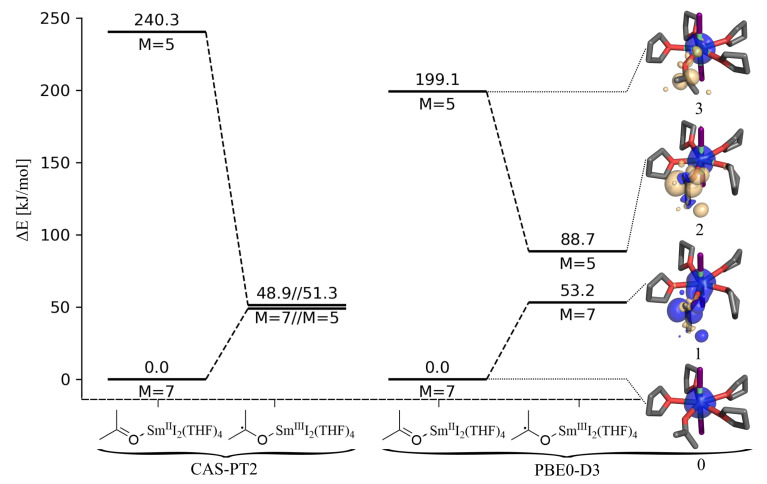
Single-point energies in (kJ/mol) for the structures ACE-Sm^II^$I_{2}$${\left(\mathrm{THF}\right)}_{4}$ and ACE${}^{•}$-Sm^III^$I_{2}$${\left(\mathrm{THF}\right)}_{4}$, calculated as septet (M = 7) and quintet (M = 5), with CASPT2 (6,13) (**left**) and with PBE0-D3 together with spin difference densities (**right**). The PBE0-D3 quintet calculations show spin contamination ($\langle {\hat{S}}^{2}\rangle =7.0$ instead of 7.75).

The quintet and septet energies are shown in Figure 2 for both the acetone (ACE-Sm^II^$I_{2}$${\left(\mathrm{THF}\right)}_{4}$) and the ketyl system (ACE${}^{•}$-Sm^III^$I_{2}$${\left(\mathrm{THF}\right)}_{4}$). The CASPT2 calculation predicts large differences between the septet and quintet for the acetone system. The actual energy difference of 240 kJ/mol has to be taken with care as the $\pi^{*}$ orbital is not in the active space. However, the spin crossover energy is high for the *f*-electrons localized at the Sm core. That is totally different for the ketyl moiety, where the septet and quintet are quasi-degenerate, as expected with one electron being delocalized at the organic substrate. The degenerate states’ SET-electron shows only minor interactions with the spins at the samarium core.

We want to check whether the PBE0-D3 functional can reproduce the spin states of the systems reasonably well. For the ACE-Sm^II^$I_{2}$${\left(\mathrm{THF}\right)}_{4}$ case, there is also a large energy difference between the septet and the quintet, while in the ketyl case, the septet and the quintet are significantly closer in energy. However, the energy difference of 35.5 kJ/mol is still ten times larger then in the CASPT2 result. As the electron is not fully transferred in the DFT calculation (for further details, see Supplementary Materials SI.3), the spin flip needs more energy. Exact exchange is one possible reason for the inaccurate difference in energy between the quintet and the septet, while the spin contamination of approximately 10% may also impact the energy as well as a self-interaction error. Although the CASPT2 picture of the SET is not perfectly reproduced, PBE0-D3 describes the situation surprisingly well.

### 2.4. Effects of HMPA as Cosolvent

HMPA is a commonly used cosolvent in $SmI_{2}$-mediated reactions. The cosolvent replaces THF molecules and increases the reduction potential [28,34,37,38]. We model a second solvation shell via the implicit solvent model COSMO with a dielectric constant resembling THF. The dielectric constant of HMPA is not used because the cosolvent HMPA is bound to samarium in experiments with [HMPA] ≤ 4 eq. The comparison experiment needs further validation as to whether the structures obtained with large-core ECP are sufficiently accurate. Therefore, we repeat the structure optimizations with ECP28MWB instead of ECP52MWB and with the use of the COSMO model in this section.

The herein used $SmI_{2}{\left(\mathrm{HMPA}\right)}_{4}$ approximates the experimentally predicted solvent-separated ion pair $\left[Sm{\left(\mathrm{HMPA}\right)}_{4}{\left(\mathrm{THF}\right)}_{2}\right]I_{2}$ [33]. According to experiments, the reduction power of $SmI_{2}$ increases when ions are displaced from the metal center [39]. A realistic study of an ion-separated system needs explicit free solvent molecules, as they are used in closed shell molecular dynamic studies of Ramirez et al. [19]. Furthermore, Ramirez applied periodic boundary conditions. They are needed to obtain the correct placement of all solvent molecules and the free iodine. However, such calculations can only be carried out with large core potentials. To be able to model the SET process, we employed a small-core ECP within non-periodic DFT calculations. Periodic boundary conditions are not feasible with this small-core ECP. Hence, the neutral $SmI_{2}{\left(\mathrm{HMPA}\right)}_{4}$ gives the best achievable model for the realistic system, which allows us to study the SET energy in a good approximation.

**Figure 3:**
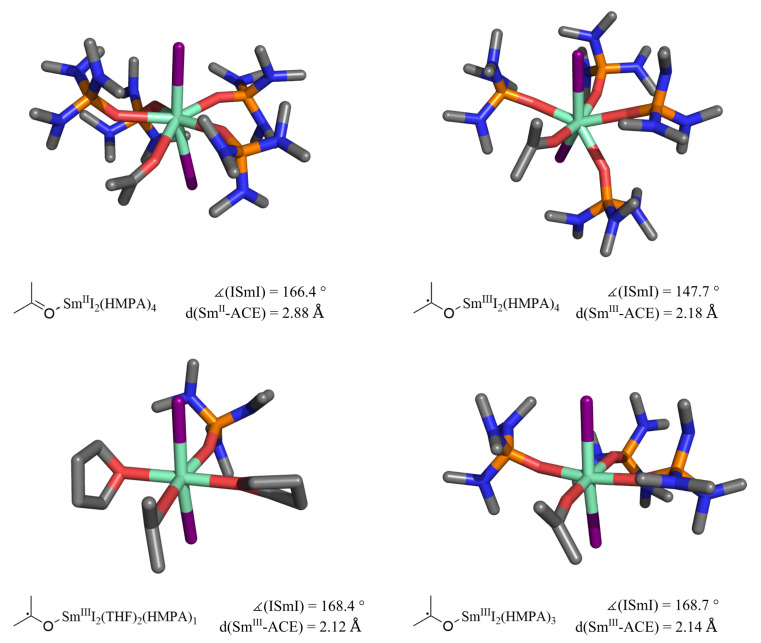
The ACE-Sm^II^$I_{2}$${\left(\mathrm{HMPA}\right)}_{4}$ structure is optimized with PBE0-D3/def2-TZVP (H, C, N, O, P, I) and ECP52MWB-II (Sm), while ECP51MWB-SV (Sm) is used for optimization of ACE${}^{•}$-Sm^III^$I_{2}$${\left(\mathrm{THF}\right)}_{2}$${\left(\mathrm{HMPA}\right)}_{1}$, ACE${}^{•}$-Sm^III^$I_{2}$${\left(\mathrm{HMPA}\right)}_{3}$ and ACE${}^{•}$-Sm^III^$I_{2}$${\left(\mathrm{HMPA}\right)}_{4}$. ${∡}_{ISmI}$ indicate the structural change in ACE${}^{•}$-Sm^III^$I_{2}$${\left(\mathrm{HMPA}\right)}_{4}$.

$SmI_{2}{\left(\mathrm{HMPA}\right)}_{4}$ bound to acetone is used as the basis for the structures in this section. A total of eight structures were obtained from ACE-Sm^II^$I_{2}$${\left(\mathrm{THF}\right)}_{4}$, ACE-Sm^II^$I_{2}$${\left(\mathrm{THF}\right)}_{2}$${\left(\mathrm{HMPA}\right)}_{1}$, ACE-Sm^II^$I_{2}$${\left(\mathrm{HMPA}\right)}_{3}$, ACE-Sm^II^$I_{2}$${\left(\mathrm{HMPA}\right)}_{4}$ and the corresponding structures of ACE-Sm^III^${}^{•}$$I_{2}$${\left(\mathrm{THF}\right)}_{m}$(HMPA)_n_, for which examples are shown in Figure 3. The bond length shortening of the SET step causes a structural distortion for ACE${}^{•}$-Sm^III^$I_{2}$${\left(\mathrm{HMPA}\right)}_{4}$ compared to ACE-Sm^II^$I_{2}$${\left(\mathrm{HMPA}\right)}_{4}$ in the upper part of Figure 3. There, the fourth HMPA ligand is pushed out of plane, which bends the ISmI angle. Therefore, we introduced sixfold coordinated structures, which are seen as distortion-free. The ACE-Sm$I_{2}$${\left(\mathrm{HMPA}\right)}_{3}$ structure reflects the substitution of one HMPA molecule by acetone. HMPA is in the *trans*-position to the acetone molecule in $\mathrm{ACE}-SmI_{2}{\left(\mathrm{THF}\right)}_{2}\left(\mathrm{HMPA}\right)$ since we expect the highest influence on the binding in this position by the polarization of the Sm-ACE bond. The structures with a coordination number of 6 do not show distortion through SET, which is highlighted by ${∡}_{ISmI}$.

We compare the changes in the optimized structures of the well-studied benzophenone system reported by Kefalidis et al. [35] to our system in the following. Structure optimization is reported with B3PW91 instead of PBE0-D3 and explicit THF molecules only. The application of COSMO elongates their SmI bond lengths by 0.38 Å, which is significantly larger than our change of 0.06 Å. This can be rationalized with our use of dispersion correction. The reoptimization of ACE-Sm^II^$I_{2}$ structures with ECP28MWB causes low structural changes, which are also reported [35]. The substitution of all THF by four HMPA leads to an increased Sm-I bond length of 0.2 Å, which is in agreement with the crystal structures [32]. The same substitution leads to changes in the Sm-ACE bond length of 0.05 Å.

Table 2 shows the influence of HMPA on the SET energies and the highest singly occupied molecular orbital (SOMO). Additionally, the Table presents results by different optimizations (ECP28MWB instead of ECP52MWB) and with additional implicit solvation.

The optimization with small-core potentials decreases the energy of ACE-Sm^II^$I_{2}$ by 10 kJ/mol. One exception is the molecule with four HMPA ligands, which is less stabilized. The replacement of two THF by one HMPA molecule has an effect on the reduction potential only when COSMO is applied. Implicit solvation shifts the equilibrium towards ACE-Sm^II^$I_{2}$ and increases the effect of HMPA on the SET, which is further discussed in the Supplementary Materials SI.4. It can be seen from the differences between $SmI_{2}{\left(\mathrm{THF}\right)}_{4}$ and $SmI_{2}{\left(\mathrm{THF}\right)}_{2}{\left(\mathrm{HMPA}\right)}_{1}$ that COSMO has a special effect on the 6-fold coordinated structure. Two small THF molecules and two other ligands form the solvent accessible surface (SAS) of the implicit solvation model. The samarium atom is shielded by the explicit solvent environment, as is indicated by electron surface potential maps in the Supplementary Materials SI.4. The application of COSMO for optimization and SET energy calculation significantly increases the total influence of HMPA.

Four HMPA ligands have a lower influence on the SET energy than three HMPA ligands in most cases accourding to our calculations. Three HMPA ligands have a higher influence because the sterical effects of the SET are unhindered. As mentioned earlier, the SET leads to a contraction of the ligand shell. Sterical effects can be used to explain the non-linear concentration dependence on HMPA reported by Shabangi et al. [34]. Less bulky solvents with similar electronic properties have the best chances to replace HMPA as a cosolvent.

Let us assume that HMPA influences the ionization potential similarly to the reduction potential. We can then approximate the influence on the reduction potential by the sign-inverted SOMO energy according to Koopmans Theorem. The SOMO energies of ACE-Sm^II^$I_{2}$ structures are given in Table 2. Optimization with a small-core ECP is necessary to interpret the SOMO energies. Therefore, the values of $\epsilon_{P52,g}$ are not interpreted. The application of COSMO shifts SOMO energies constantly by about −25 kJ/mol. The SOMO energy change of ACE-Sm^II^$I_{2}$${\left(\mathrm{HMPA}\right)}_{4}$ to ACE-Sm^II^$I_{2}$${\left(\mathrm{THF}\right)}_{4}$ is 89 kJ/mol, which reasonably reproduces the magnitude of linear sweep experiments. The change from ACE-Sm^II^$I_{2}$${\left(\mathrm{HMPA}\right)}_{3}$ to ACE-Sm^II^$I_{2}$${\left(\mathrm{THF}\right)}_{4}$ is 67 kJ/mol, which is also reasonable compared to the experimental value of 59.8 kJ/mol (3 eq. HMPA). The difference towards the experiment is surprisingly small when we think about the approximations and about the fact that the discussed energies come from Kohn–Sham orbitals. Hence, this shows that our DFT results reproduce the HMPA effect in agreement with measurements coming from linear sweeps as well as the one from the rate experiments [28,34].

## 3. Methodology

Unrestricted single-determinant Kohn–Sham DFT calculations were performed using the TURBOMOLE 7.3 program package. Dispersion correction with Becke Johnson damping was applied and noted explicitly or by the sufix -D3 for the respective functionals. All structures were optimized with the PBE0-D3/def2-TZVP basis set with corresponding ECP28MWB for iodine and the basis sets ECP52MWB-II [22,40,41] (Sm^II^) and ECP51MWB-SV [22,23] (Sm^III^) in conjunction with the corresponding large-core Stuttgart–Dresden ECPs. Single-point energies were calculated with the def2-TZVP basis set with ECP28MWB for samarium and iodine. The density functional benchmark includes single-point energy calculations with the following functionals: PBE0 [42,43,44,45,46], B3LYP [42,43,47,48,49,50], BHLYP [42,43,48,49,51], PBE [42,43,44,45], TPSS [42,43,44,52], TPSSH [42,43,44,52,53], and B3PW91 [42,43,45,48]. The exchange correlation functionals were integrated with multigrid m4. The thermal smearing of electrons improved the initial wavefunction guess of single-point energy calculations. The SCF energy convergence threshold was $10^{-6}$au, and gradients were converged to $10^{-3}$au.

In a second part of the study, the described PBE0-D3/def2-TZVP optimization with large-core ECPs was applied for the further investigation of the influence of HMPA. Within this section, large-core optimization was compared to small-core optimization. For this purpose, the large-core ECP52MWB was exchanged by the small-core ECP28MWB for simulating Sm^II^. The application of COSMO [54] (ϵ=7.4, THF) in single-point calculations and structure optimization was also evaluated. Other parameters were used as described above.

Complete active space self-consistent field (CASSCF) single-point calculations were performed for the DFT-optimized structures using the OpenMolcas program (V18.09, pymolcas version 2.05) [55] in the gas phase. An active space with thirteen orbitals and their corresponding six electrons was employed, denoted (6,13). The active orbitals identified approximately as seven 4*f* orbitals and six 5*f* orbitals for polarization when the Sm^II^ species was calculated. The Sm^III^ species’ active space included seven 4*f* orbitals, five 5*f* orbitals and the π* orbital of the organic environment. The all-electron ANO-RCC basis sets were used with the following contractions: 8s7p4d3f2g1h for Sm, 4s3p2d1f for O, 4s3p2d1f for C, 7s6p4d2f1g for I, and 1s for H [56,57]. Scalar relativistic effects were included with the second-order Douglas–Kroll–Hess Hamiltonian (DKH2). The cost of integral evaluation was reduced using Cholesky decomposition in combination with local exchange screening. For both complexes, the septet and quintet states were calculated. Spin-splitting energies were reported for second-order multireference perturbation theory (CASPT2). In the CASPT2 zeroth-order Hamiltonian, both an imaginary shift of 0.2 au and an IPEA shift of 0.25 were employed.

## 4. Conclusions

SmI${}_{2}$-induced electron transfer is presented for the acetone-ketyl equilibrium of ACE-Sm^II^$I_{2}$ and ACE${}^{•}$-Sm^III^$I_{2}$ in explicit THF solvent under CASPT2 and various DFT methods. Furthermore, the effect of the cosolvent HMPA on the single electron transfer (SET) was modelled by replacing THF with explicit HMPA molecules.

The single-point CASPT2 calculation shows an endotherm value for the electron transfer, such that the aceton-ketyl radical equilibrium lies on the side of the acetone. The predicted equilibrium reaction is in accordance with postulates [28].

The benchmark calculation with different DFT functionals validates the choice of the PBE0-D3 functional as a good one for DFT studies involving $SmI_{2}$ redox reactions and confirms its application in previous work [16]. PBE0-D3 reproduces the CASPT2 (6,13) energy difference of the septet state quantitatively. The large gap between the ACE-Sm^II^$I_{2}$${\left(\mathrm{THF}\right)}_{4}$ quintet and septet tells us that the quintet is not involved in the SET on the ACE-Sm^II^$I_{2}$${\left(\mathrm{THF}\right)}_{4}$ side of the reaction. An almost degenerate quintet/septet state of the ACE${}^{•}$-Sm^III^$I_{2}$${\left(\mathrm{THF}\right)}_{4}$ structure is foretold by the CASPT2 calculation. The degenerate state is not as well reproduced by PBE0-D3 because of spin contamination, self-interaction and/or overestimated exchange energy. Hence, SET reactions need further investigations for unambiguous statements as to the DFT picture of samarium diiodide reactions.

The influence of HMPA on the acetone-ketyl equilibrium is estimated by the replacement of explicit THF molecules with HMPA. We could rationalize the different values of the influence of HMPA obtained from experiments. The strong influence of HMPA on the reduction potential [34] can be explained by the destabilization of the SOMO, whereas the influence on the SET energy reflects the effects on rates [28]. HMPA influences the equilibrium and increases the stability of the ketyl radical, as was postulated by Curran and coworkers [28].

Overall, our results show the possibilities and limits of the PBE0-D3 functional for the attempt to describe $SmI_{2}$-mediated reactions and how they can be influenced by HMPA.

## Figures and Tables

**Table 1:** Gas phase SET energies are shown in (kJ/mol) ($\Delta E=E_{{\mathrm{ACE}}^{•}-\mathrm{Sm}\mathrm{III}I_{2}{\left(\mathrm{THF}\right)}_{4}}-E_{\mathrm{ACE}-\mathrm{Sm}\mathrm{II}I_{2}{\left(\mathrm{THF}\right)}_{4}}$). CAS (6,13), and DFT energies are calculated as described in the methodology section. Structures ACE-Sm^II^$I_{2}$ and ACE${}^{•}$-Sm^III^$I_{2}$ are optimized with ECP52MWB-II and ECP51MWB-SV, respectively, and the PBE0-D3 functional in gas phase.

	ΔE
CASSCF (6,13)	51.94
CASPT2 (6,13)	48.94
TPSS-D3	12.76
PBE-D3	28.91
TPSSH-D3	27.28
PBE0-D3	53.19
B3LYP-D3	55.40
BHLYP-D3	38.42
B3PW91-D3	69.66
B3PW91	40.02

**Table 2:** SET energies $\Delta E=E_{{\mathrm{ACE}}^{•}-{\mathrm{Sm}}^{\mathrm{III}}I_{2}}-E_{\mathrm{ACE}-{\mathrm{Sm}}^{\mathrm{II}}I_{2}}$ are calculated for different numbers and types of solvents with PBE0-D3/def2-TZVP. $\Delta E_{P52,g}$ is calculated with the usual methodology. $\Delta E_{P28,g}$, $\Delta E_{P28,g}$ and $\Delta E_{P28,solv}$ use ECP28MWB instead of ECP52MWB for Sm in the structure optimization of ACE-Sm^II^$I_{2}$, while ACE${}^{•}$-Sm^III^$I_{2}$ structures are optimized with ECP51MWB in both the gas phase (*g*) and with the COSMO model of solvation (solv). For $\Delta E_{P28,solv}$, implicit solvation is used in structure optimization as well as single-point calculation of ACE-Sm^II^$I_{2}$ and ACE${}^{•}$-Sm^III^$I_{2}$. SOMO energies (ϵ) are shown for ACE-Sm^II^$I_{2}$ and the herein-mentioned methodology.

Molecule	$\Delta E_{P52,g}$	$\Delta E_{P28,g}$	$\Delta E_{P28,\mathrm{solv}}$	$\epsilon_{P52,g}$	$\epsilon_{P28,g}$	$\epsilon_{P28,\mathrm{solv}}$
$SmI_{2}{\left(\mathrm{THF}\right)}_{4}$	53.19	66.92	84.61	−331.54	−335.73	−359.60
$SmI_{2}{\left(\mathrm{THF}\right)}_{2}{\left(\mathrm{HMPA}\right)}_{1}$	52.74	65.80	77.70	−333.64	−336.55	−357.36
$SmI_{2}{\left(\mathrm{HMPA}\right)}_{3}$	37.50	52.74	58.14	−262.95	−262.06	−292.57
$SmI_{2}{\left(\mathrm{HMPA}\right)}_{4}$	47.90	51.56	63.26	−270.83	−245.93	−271.51

## Data Availability

See Supplementary Materials. Further data can be requested from the authors.

## Supplementary Materials

The following supporting information can be downloaded at: https://www.mdpi.com/article/10.3390/molecules27248673/s1, Section S.1: Comparison of bound and unbound acetone and ketyl radical anions; Section S.2 The benchmark CAS calculation; Section S.3 Benchmark for DFT functionals against the CASPT2(6,13) calculation; Section S.4 Electron surface potentials; Cartesian coordinates of the PBE0-D3 optimized geometries in .xyz format.

## Abbreviations

The following abbreviations are used in this manuscript:

DFT	Density functional theory
SET	Single electron transfer
ACE	Acetone
ACE${}^{-•}$	Ketyl radical anion
ECP	Electron core potential
