# Supplementary material for: Samarium Diiodide Acting on Acetone—Modeling Single Electron Transfer Energetics in Solution
Source: Molecules. 2022 Dec 8;27(24):8673. doi: 10.3390/molecules27248673 (PMC9781745; doi:10.3390/molecules27248673)
Supplement: Supplementary file 1 [file molecules-27-08673-s001.zip › molecules-2050633 supplementary.pdf]

Supplementary Materials: Samarium Diiodide Acting on Acetone — Modeling Single Electron Transfer Energetics in Solution

Luca Steiner, Andreas J. Achazi, Bess Vlaisavljevich, Pere Miro, Beate Paulus and Anne-Marie Kelterer

Contents

1. Comparison of the bound and unbound Acetone and Ketyl Radical Anion . . . 1
2. The benchmark CAS calculation . . . 1
3. Benchmark for DFT functionals against the CASPT2(6,13) calculation . . . 2
4. Electron surface potentials . . . 3

1. Comparison of the bound and unbound Acetone and Ketyl Radical Anion

Figure S1 shows the structure of the free ketyl radical anion and aceton. Structural parameters of the bound and the free $CH_3{-}CO{-}CH_3$ moiet are found in Table S1 for the bound and free acetone and ketyl radical. The C=O distance changes by 0.08 Å when the free acetone structure is compared to the ketyl radical anion structure. The distance $d_{C_1C_2}$ does not change significantly in this case. The dihedral angle $\phi_{C_1C_2OC_3}$ reflects the planarity of the acetone and non-planarity of the ketyl radical anion. The parameters have similar values compared to the same parameters in the bound case of ACE-$Sm^{II}I_2(THF)_4$ and ACE$^\bullet$-$Sm^{III}I_2(THF)_4$. One interesting difference is the further elongation of the carbonyl bond in the bound case, which indicates that the reduced carbonyl bond is destabilized by coordination of Sm. This destabilization of the carbonyl bond may come from an overall stabilizing electron back donation towards the metal center when a ketyl radical anion gets bound to a formal $Sm^{III}I_2^+$ species. However, the part of the structure of ACE$^\bullet$-$Sm^{III}I_2(THF)_4$ is very similar to a ketyl radical and will be named as one in this work. The structural parameters change similarly which again supports that PBE0-D3 describes the SET mediated by SmI_2 well.

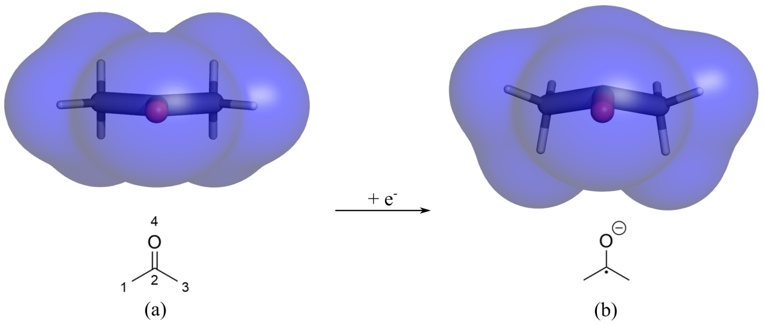

Figure S1. Reduction of acetone (left) to the ketyl radical (right) is shown with total density . Contour value for the density is 0.002 au. Structures are optimized with PBE0/def2-TZVP in gas phase.

2. The benchmark CAS calculation

The CAS calculations are discussed in more detail in this section including dynamic correlation and pertubation theory, quintet and septet states, SET energy and the small or large CAS space. The results of the CAS calculations are shown in Table S2.

Table S1. Characteristic bond lengths in [Å], angles and dihedral angles in [°] are compared between the bound and unbound acetone and ketyl radical structure. The atom numbering is shown in Figure S1(a).

Parameter	ACE	$ACE^{-\bullet}$	ACE-$Sm^{II}I_2(THF)_4$	$ACE^{\bullet}$-$Sm^{III}I_2(THF)_4$
$d_{C_2O_4}$	1.21	1.29	1.21	1.33
$d_{C_1C_2}$	1.51	1.51	1.50	1.49
$\phi_{C_1C_2O_4C_3}$	180.0	142.8	179.7	150.7

Table S2. Single point energies of quintet (M=5) and septet (M=7) state of ACE-$Sm^{II}I_2(THF)_4$ and $ACE^{\bullet}$-$Sm^{III}I_2(THF)_4$ calculated with ANO-RCC-VTZP (Sm), ANO-RCC-VDZP (C,O,I), ANO-RCC-MB (H) basis sets in [kJ/mol] are compared between two complete active spaces (CAS) and correction from second order perturbation theory (PT2). ACE-$Sm^{II}I_2(THF)_4$ is chosen as zero point of energy for different methodologies.

		CASSCF		CASPT2	
CAS	M	Sm^{II}	Sm^{III}	Sm^{II}	Sm^{III}
(6,13)	7	0.00	51.94	0.00	48.94
(6,13)	5	275.81	53.52	240.27	51.29
(6,8)	7	0.00	-10.34	0.00	69.66
(6,8)	5	293.62	-9.72	310.16	70.22

The CAS space of ACE-$Sm^{II}I_2(THF)_4$ includes six singly occupied $4f$ orbitals, one unoccupied $4f$ orbital and six unoccupied $5f$ orbitals. $5d$ orbitals are not included in the calculation. The CAS space of $ACE^{\bullet}$-$Sm^{III}I_2(THF)_4$ includes one singly occupied π^* orbital of the acetone molecule, five singly occupied $4f$ orbitals, two unoccupied $4f$ orbitals and five unoccupied $5f$ orbitals. If we use a CAS(6,13) space, the SET energy is 51.94 kJ/mol, and one electron is completely transferred in the reduction step. The CASSCF and CASPT2 SET energies differ only by 3 kJ/mol. Thus, the SCF level of the CAS space already includes a significant part of dynamical correlations.

That is different with the smaller CAS (6,8) space. The ACE-$Sm^{II}I_2(THF)_4$ structure is calculated with six $4f$ orbitals and two $5f$ orbitals, while the $ACE^{\bullet}$-$Sm^{III}I_2(THF)_4$ structure is calculated with seven $4f$ orbitals and the π^* orbital. Although the CAS(6,8) space is similar, $5f$ orbitals are missing. There, the CASSCF gives an exothermic charge transfer, whereas including dynamical correlations at the PT2 levels again results in an endothermic reaction, which is even larger as the CAS(6,13) treatment. Hence, the $5f$ orbitals contribute significantly to dynamical effects and should be included in the CAS calculation.

The CAS(6,8) quintet $ACE^{\bullet}$-$Sm^{III}I_2(THF)_4$ experiences an effect of the same magnitude from PT2.

The quintet of the CAS(6,13) calculation is calculated with the septet as initial guess. The active space stays the same as the one for the septet. From Figure 2 of the main article it can be told that the π^* orbital may contribute to energy of the quintet of ACE-$Sm^{II}I_2(THF)_4$. Therefore, the inclusion of dynamical correlation at the PT2 level changes the result significantly with -35.54 kJ/mol. $ACE^{\bullet}$-$Sm^{III}I_2(THF)_4$ quintet accounts well for dynamic correlation since the π^* is properly included, which can be seen by the small impact of PT2 of 2.23 kJ/mol.

3. Benchmark for DFT functionals against the CASPT2(6,13) calculation

The influence of dispersion on the SET energies is discussed in comparison with the reference CASPT2 result together with the amount of electron transfer. In Table S3, charges, orbital occupation and energies of $ACE^{\bullet}$-$Sm^{III}I_2(THF)_4$ relative to ACE-$Sm^{II}I_2(THF)_4$ are compared with and without dispersion correction. D3-corrected energies are discussed in the results section. When dispersion correction is excluded, it can be seen that the

functionals TPSSH and PBE are close to the reference CASPT2 energy. However, dispersion interaction is high (e.g. 16.5 kJ/mol for PBE0) and cannot be ignored for the main reaction path. In particular, long range contributions cannot be neglected for the explicit solvent environment and it usually is important for structure optimization.

f-orbital occupation numbers show that one electron is transferred in the CAS calculation. DFT computes only a partial electron transfer at Sm. PBE0 and B3LYP transfer 0.5 e, whereas the other functionals transfer only ca. 0.3 e. Thus, the f orbital occupation gets better when exact exchange is included. Partial charges are generally not so well represented by the DFT calculations and are not discussed in detail, for this reason. Nevertheless, hybrid functionals show the correct trend of charge increase upon reduction, while non-hybrid functionals have a reversed trend which makes no physical sense.

Table S3. Single point SET energies are shown in [kJ/mol] ($\Delta E = E_{ACE^{\bullet}\text{-}Sm^{III}I_2(THF)_4} - E_{ACE\text{-}Sm^{II}I_2(THF)_4}$). For DFT functionals, ΔE_{noDisp} depicts energies without dispersion correction, and ΔE includes electronic energy and dispersion correction. f orbital occupation numbers ($n_{occ,f}$) and charges at Sm (q_{Sm}) come from natural bond order analysis in the DFT method and from Mulliken population analysis in the CAS method. The first lines show the CASSCF(6,13) SET energy and the CASPT2 energy.

	ΔE	ΔE_{noDisp}	$n_{occ,f}$		q_{Sm}	
			Sm^{II}	Sm^{III}	Sm^{II}	Sm^{III}
CASSCF (6,13)	51.94		6.00	5.02	1.94	2.41
CASPT2 (6,13)	48.94					
PBE	28.91	46.03	5.90	5.62	0.95	0.90
PBE0	53.19	69.65	6.02	5.49	1.01	1.04
B3LYP	55.40	85.89	6.01	5.51	1.13	1.23
BHLYP	38.42	63.80	6.03	5.17	1.20	1.41
B3PW91	69.66	40.02	5.91	5.62	1.03	1.01
TPSS	12.76	35.67	5.90	5.59	0.99	0.94
TPSSH	27.28	49.30	5.96	5.56	1.01	1.00

4. Electron surface potentials

Electrostatic surface potentials (ESP) in Figure S2 show that the ligands fully shield Sm. Hence, the impact of an implicit solvent model like COSMO describes the influence of the second solvation shell, which is quite small. The positively charged hydrogen atoms of the acetone molecule of ACE-$Sm^{II}I_2(THF)_4$ gets neutral when another electron is located in $ACE^{\bullet}$-$Sm^{III}I_2(THF)_4$. The effect is small and can be seen well at the acetone – ketyl radical moiety. The more HMPA ligands are in the structure, the lower gets the effect. The samarium atom in a higher oxidation state slightly decreases the negative charge at the electrostatic potential surface of the iodine atoms. Curiously, one could think, that $ACE^{\bullet}$-$Sm^{III}I_2(THF)_4$ shows the higher charge on the surface but the explicit solvent environment inverts it and the surface charge decreases when Sm^{III} is formed. Since these surfaces are less charged in $ACE^{\bullet}$-$Sm^{III}I_2(THF)_4$, it is stabilized by COSMO less than ACE-$Sm^{II}I_2(THF)_4$.

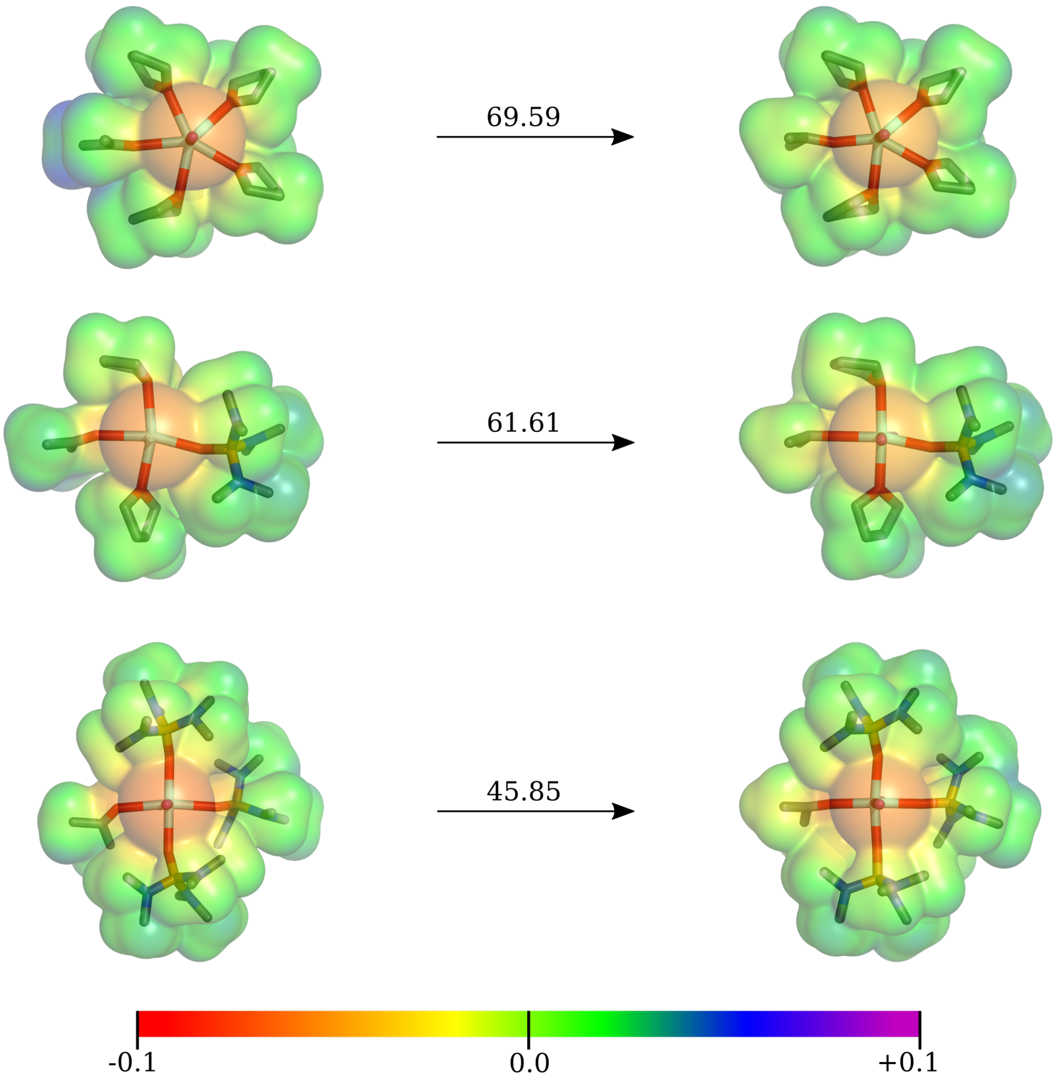

Figure S2. ACE-$Sm^{II}I_2$ (left) and ACE$^{\bullet}$-$Sm^{III}I_2$ (right) with 0/3 (top), 1/2 (center), 3/0 (bottom) HMPA/THF ligands. Electrostatic potential maps in E_h/e are mapped on a constant density of 0.002. ESPs are calculated with PBE0-D3/def2-TZVP with ECP28MWB (Sm) and COSMO.
